# Postpartum Serosal Eosinophilic Gastroenteritis: A Rare Cause of Ascites

**DOI:** 10.7759/cureus.96477

**Published:** 2025-11-10

**Authors:** Anas Hatab, Rawan Abouhatab, Hassan Hatab

**Affiliations:** 1 Gastroenterology, Royal Blackburn Teaching Hospital, Manchester, GBR; 2 General Medicine, Lancashire Teaching Hospitals NHS Foundation Trust, Preston, GBR; 3 Gastroenterology, East Lancashire Hospitals NHS Trust, Blackburn, GBR

**Keywords:** corticosteroid responsiveness, eosinophilic ascites, eosinophilic gastroenteritis, postpartum, serosal subtype

## Abstract

Eosinophilic gastroenteritis (EGE) is a rare, heterogeneous inflammatory disorder of the gut wall. Eosinophils are recruited to one or more layers of the gastrointestinal wall. The serosal type is uncommon and can mimic other causes of ascites, such as spontaneous bacterial peritonitis (SBP) or malignancy. We report the case of a woman in her early 30s who presented six weeks postpartum with epigastric pain and recurrent vomiting. She was discharged with oral medications, which did not settle her, resulting in readmission to the hospital. Here, her blood demonstrated marked eosinophilia with eosinophils rising to 8.2 × 10⁹/L. Computed tomography (CT) scan showed ascites and diffuse thickening of the small bowel wall. Cytology of the ascitic fluid revealed eosinophil dominance. No other pathology was identified. Oesophagogastroduodenoscopy (OGD) showed duodenal erythema. A diagnosis of serosal EGE was made based on eosinophilic ascites, small-bowel thickening, and her rapid improvement after treatment with steroids. This case highlights key learning points. Firstly, serosal EGE can occur with normal mucosal biopsies. Secondly, the presence of neutrophilia in the ascitic fluid does not always indicate SBP. Lastly, steroid therapy can be both diagnostic and therapeutic.

## Introduction

Eosinophilic gastroenteritis (EGE) is an unusual, heterogeneous, inflammatory condition of the gastrointestinal tract, characterised by the accumulation of eosinophils in one or more layers of the gut wall. It usually presents in the absence of secondary causes, such as parasitosis, drug-induced hypersensitivity, or systemic disorders [1]. Its symptomology is variable and primarily determined by the extent of tissue involved, which can further contribute to the diagnostic enigma [2].

The mucosal form of EGE is the most common and typically presents with abdominal pain, diarrhoea, malabsorption, or even gastrointestinal bleeding [3]. By contrast, involvement of the muscularis propria in the muscular type may lead to bowel wall thickening and obstructive symptoms [4]. The serosal form, which is the least frequently observed, usually presents with eosinophil-rich exudative ascites and peripheral eosinophilia; it can be associated with bowel wall oedema without vasculitis [5]. Correct diagnosis requires ruling out other causes of eosinophilic ascites, such as malignancy, infection, and autoimmune disease [6]. Patients with serosal EGE generally respond well to corticosteroids, which remain the mainstay of management [7].

We describe the case of a young woman in the early post-partum period, who developed serosal-type EGE, highlighting the diagnostic pitfalls associated with eosinophilic ascites, the limitations of superficial endoscopic biopsies, and the importance of corticosteroid responsiveness.

The postpartum period represents a time of significant immunological transition, during which a shift from the Th2-dominant immune profile of pregnancy toward a Th1/Th17-dominant state may precipitate inflammatory or autoimmune conditions. This physiological change may act as a trigger for EGE in susceptible individuals, as illustrated by the present case.

## Case presentation

A woman in her early 30s, six weeks postpartum following an emergency caesarean section for preterm delivery, initially presented to the emergency department with a one-week history of epigastric pain and recurrent vomiting. Her obstetric history was significant for pre-eclampsia in a previous pregnancy. The patient did not take any regular medication. She had no allergies, apart from a documented hypersensitivity to ibuprofen, and reported no tobacco or alcohol use. There was no relevant personal or familial history of atopy.

On initial clinical examination, mild tachycardia was noted (pulse rate 103 beats per minute), with normal blood pressure, temperature, and oxygen saturation. Physical examination revealed mild epigastric tenderness without any signs of peritonism. Examination of the cardiovascular and respiratory system was unremarkable.

Initial blood tests revealed eosinophilia (eosinophil count: 4.9 × 10^9^/L) and normocytic anaemia. Other laboratory investigations, such as inflammatory markers, renal and liver function tests, were normal. She was provisionally diagnosed with gastritis and commenced on a treatment regimen with the prescription of proton-pump inhibitors and antiemetics. She was thus discharged with an outpatient prescription for lansoprazole.

The patient’s symptoms worsened over the subsequent fortnight, with persistent vomiting and abdominal pain. Peripheral eosinophil counts increased, peaking at 8.2 × 10⁹/L, while other blood parameters (including tumour markers and infection markers) remained normal. Further investigations to exclude secondary causes of eosinophilia, including ANCA, quantiferon-TB, β2-glycoprotein, anticardiolipin antibodies, complement levels, and galactomannan assays, were all negative.

**Figure 1:**
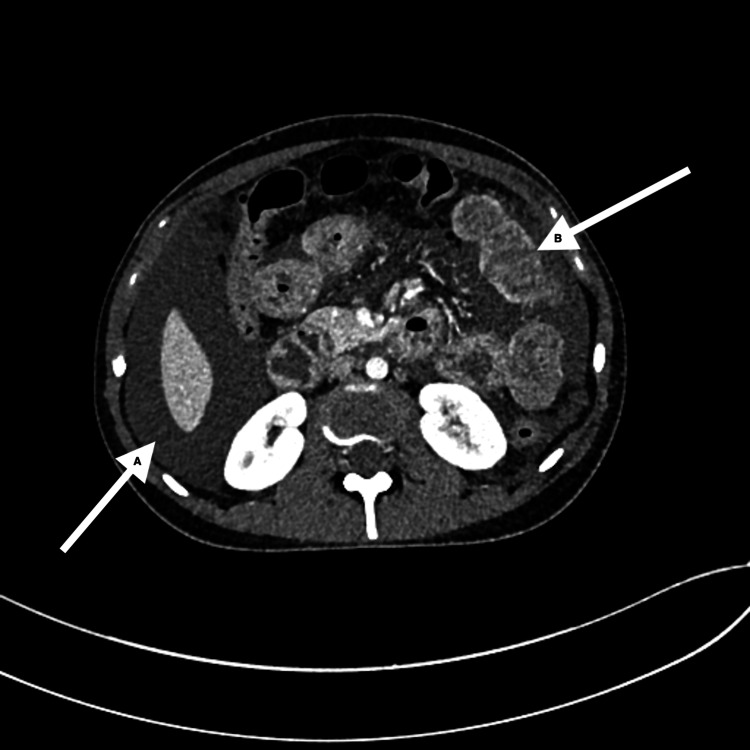
Contrast-enhanced computed tomography (CT) of the abdomen (A) The arrow demonstrates moderate ascites surrounding the liver. (B) The arrow highlights mural thickening and enhancement of small bowel loops.

Contrast-enhanced computed tomography (CT) revealed moderate ascites, diffuse mural thickening, and enhancement of multiple small bowel loops; there was no evidence of obstruction or features of portal hypertension (Figure 1).

Diagnostic paracentesis showed straw-coloured ascitic fluid containing 4.39 × 10³ eosinophils/μL and 4.00 × 10² neutrophils/μL, with a low serum-ascites albumin gradient (SAAG) of 0.6 g/dL. Empiric antibiotics were initiated for a presumed spontaneous bacterial peritonitis (SBP) based on the clinical presentation and the presence of neutrophils in the ascitic fluid, despite the eosinophilia.

**Figure 2:**
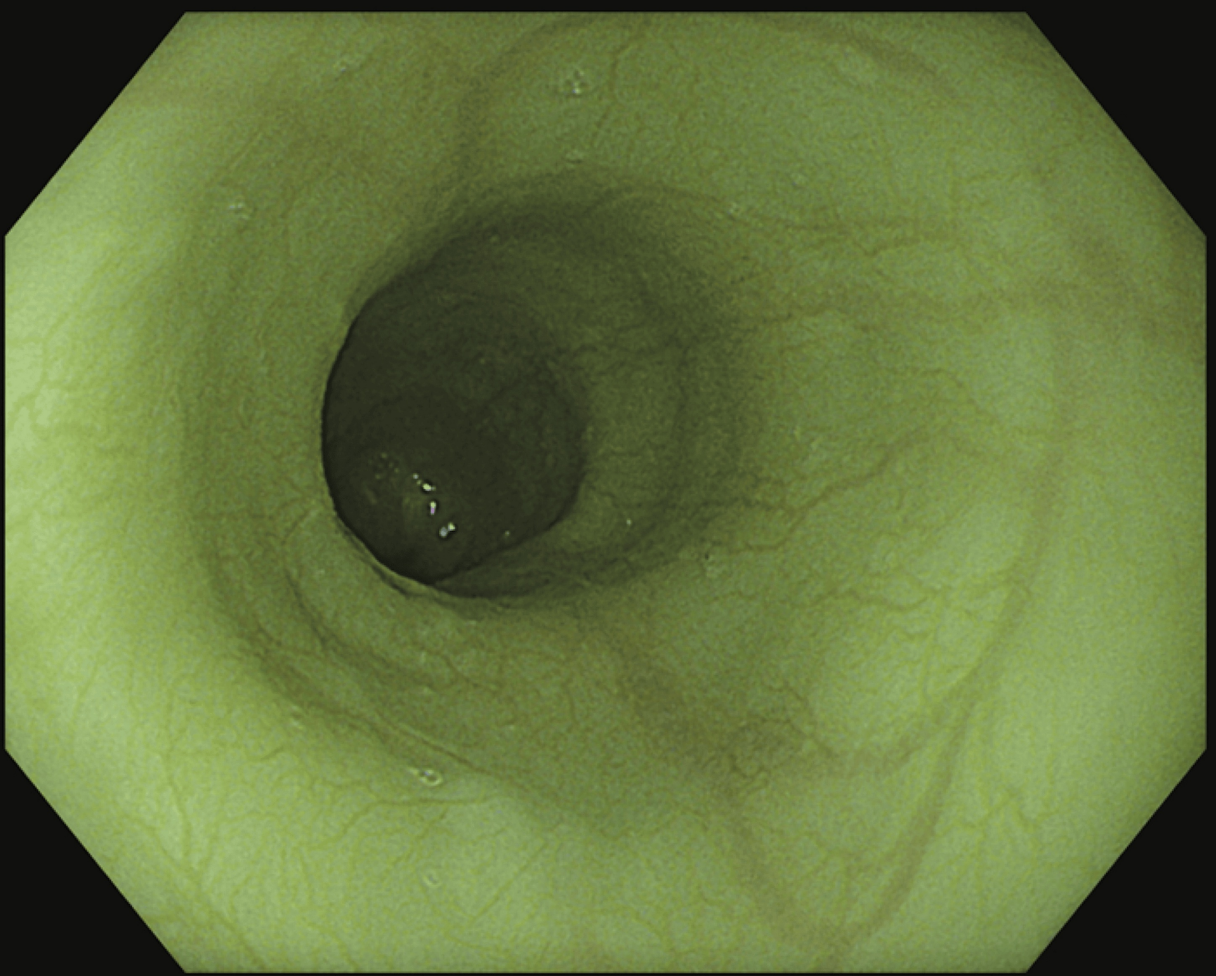
Oesophagogastroduodenoscopy (OGD) The oesophagus demonstrates prominent vascular mucosa without ulceration or mass lesion.

Oesophagogastroduodenoscopy (OGD) showed vascular mucosa, but no structural abnormalities of the stomach (Figure 2).

**Figure 3:**
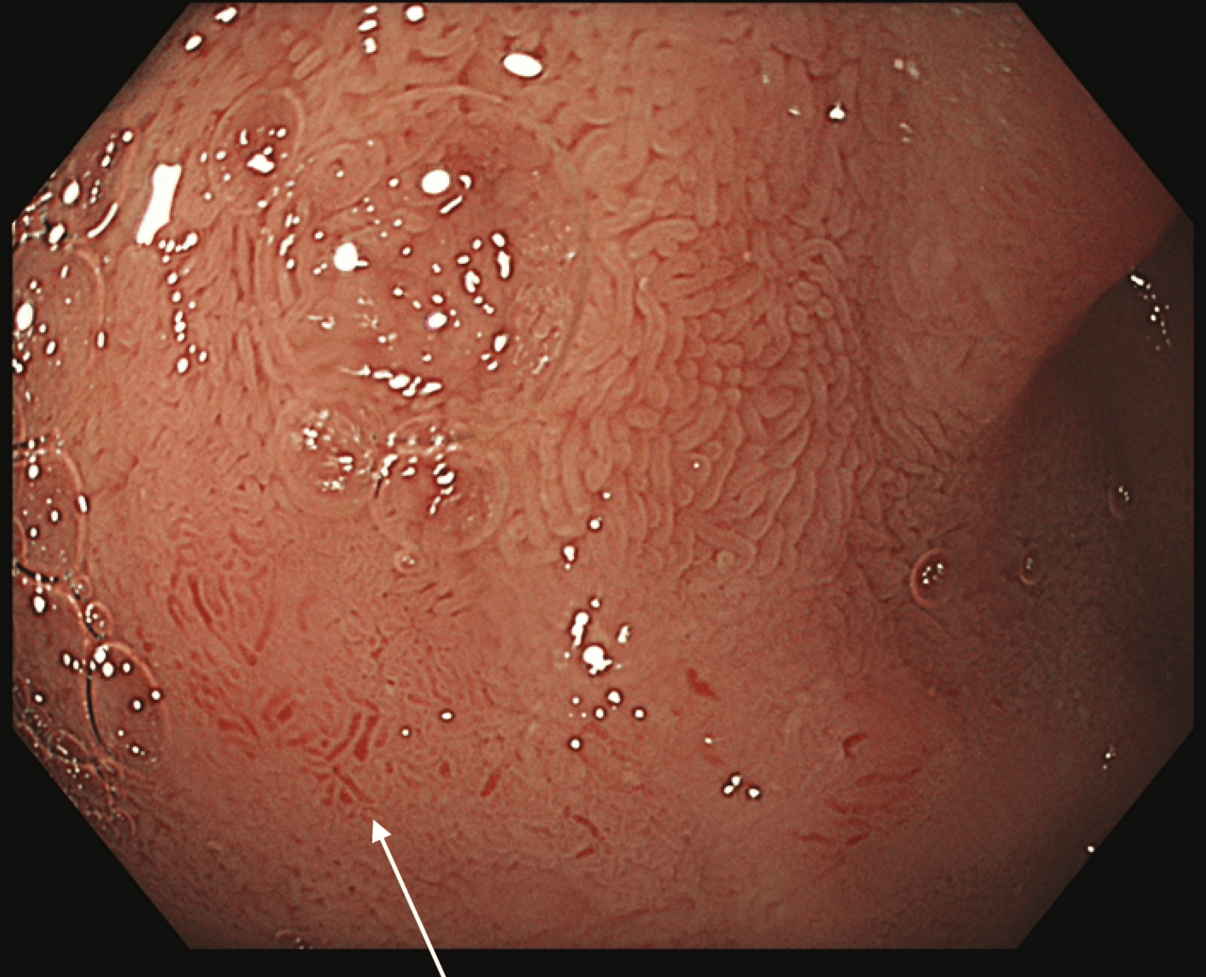
Oesophagogastroduodenoscopy (OGD) Endoscopic view of the first part of the duodenum (D1), with the arrow pointing to patchy erythema, suggestive of eosinophilic duodenitis.

In addition, the first part of the duodenum (D1) demonstrated patchy erythema (Figure 3), consistent with eosinophilic duodenitis. Histological biopsies taken from these sites were reported as normal. Video capsule endoscopy (VCE) revealed patchy erythema and granulation tissue in the stomach, along with duodenal and proximal jejunal patches of erythema and mild oedema of the villi, and loss of villous height in the proximal small bowel. 

She was commenced on oral corticosteroid therapy with prednisolone at 40 mg daily. She showed rapid symptomatic improvement and normalisation of peripheral eosinophil counts within 48 hours. 

Follow-up showed sustained clinical improvement and stable eosinophil levels. However, relapse occurred after discontinuation of steroids, accompanied by a rise in eosinophil count. Oral budesonide was initiated at 9 mg daily and tapered gradually, resulting in ongoing clinical stability, normal eosinophil counts, and weight gain at the last follow-up. On examination, the ascites had completely resolved and her abdomen was soft and non tender, three months after her second presentation.

## Discussion

EGE is characterised by eosinophilic infiltration of the gastrointestinal (GI) tract. Although the name suggests localisation to the stomach and small intestine, eosinophilic inflammation can affect any part of the GI tract, from the oesophagus to the colon. Klein et al. first proposed that eosinophilic accumulation may occur in any layer of the GI wall, mucosal, muscular, or serosal [8]. The mucosal subtype is most common, presenting with abdominal pain, diarrhoea, malabsorption, or anaemia. Muscular involvement can lead to bowel wall thickening, stricture formation, or obstruction. The serosal form is the rarest, representing fewer than 10% of EGE cases, and is characterised by eosinophilic ascites and peripheral eosinophilia [9].

The diagnosis of serosal EGE is notoriously tricky, as standard endoscopic mucosal biopsies are often non-diagnostic due to the deep-seated nature of the inflammation [8]. Our case exemplifies this challenge; the diagnosis was made from a constellation of findings, including eosinophilic ascites, significant peripheral eosinophilia, supportive radiological evidence, and a characteristic dramatic response to corticosteroid therapy [9,10]. A pivotal diagnostic pitfall in our patient was the initial misclassification of her ascites as SBP. This highlights a vital clinical lesson: SBP occurs almost exclusively in the context of advanced liver disease. In a patient with normal hepatic function, eosinophil-predominant ascites should immediately raise suspicion for alternative pathologies like serosal EGE.

Corticosteroids remain the mainstay of treatment for EGE, producing rapid symptomatic and haematological improvement in most cases [11]. However, some patients experience relapse or corticosteroid dependence. Budesonide, a locally acting steroid with lower systemic bioavailability, has been used successfully in maintenance therapy, as in our patient. In steroid-refractory or relapsing cases, biologic therapies such as omalizumab, infliximab, or ketotifen have been explored, although data remain limited.

The exact aetiology and pathogenesis of EGE remain unclear. It is thought to represent a multifactorial process involving hypersensitivity reactions to dietary or environmental antigens. Eosinophilic activation is mediated through T-helper 2 (Th2) cell pathways, interleukin-5, eotaxin, and mast cell degranulation [12-14]. Many patients have a background of atopic disease, including asthma, eczema, or drug allergies. In our case, the patient’s hypersensitivity to ibuprofen could have contributed to the eosinophilic response.

Pregnancy and the postpartum period seem to influence immune responses and may trigger eosinophilic gastrointestinal disease. During pregnancy, a Th2-dominant immune profile supports foetal tolerance, while postpartum reversion to Th1/Th17 dominance fosters inflammatory responses [15]. We hypothesise that this sudden immunological shift may trigger EGE in susceptible individuals. This case supports a possible link between postpartum immune rebalancing and the development of serosal EGE, consistent with previously reported cases in the puerperium. Our patient’s history of pre-eclampsia, a condition characterised by systemic endothelial dysfunction and immune activation, may have been an additional predisposing factor [16,17].

Fewer than ten cases of EGE associated with pregnancy or the puerperium have been reported in the literature, with only four, including the present case, describing eosinophilic ascites [17,18]. Ribeiro et al. described a 33-year-old woman who developed eosinophilic ascites seven weeks postpartum, requiring long-term corticosteroid therapy due to relapse [18]. This closely parallels our case, where symptoms recurred following steroid cessation, necessitating maintenance therapy with budesonide. Conversely, Khalil and Joseph reported a similar case with complete remission after a single steroid course [19]. This dichotomy suggests the existence of distinct disease phenotypes, an area warranting further investigation. The successful induction and maintenance of remission in our patient with budesonide highlights the utility of this agent, which offers targeted therapeutic action with a favourable systemic side-effect profile.

## Conclusions

This case adds to the limited literature on postpartum EGE, particularly the serosal subtype. Several key learning points emerge. First, serosal EGE may present with normal mucosal biopsies, highlighting the need for ascitic fluid evaluation and clinical suspicion when eosinophilic ascites is encountered. Second, not all neutrophil-rich ascitic fluid should be presumed to represent SBP in the absence of liver disease. Third, peripheral eosinophilia and a rapid response to corticosteroid therapy remain valuable diagnostic and therapeutic indicators. Finally, pregnancy and the puerperium may act as immunological triggers for EGE through postpartum immune rebalancing. Recognising this association is crucial for timely diagnosis and management, as the disease course can range from monophasic to chronic relapsing forms.

## References

[1] Méndez-Sánchez N, Chávez-Tapia NC, Vazquez-Elizondo G, Uribe M (2007). Eosinophilic gastroenteritis: a review. Dig Dis Sci.

[2] Talley NJ, Shorter RG, Phillips SF, Zinsmeister AR (1990). Eosinophilic gastroenteritis: a clinicopathological study of patients with disease of the mucosa, muscle layer, and subserosal tissues. Gut.

[3] Lucendo AJ, Arias A (2012). Eosinophilic gastroenteritis: an update. Expert Rev Gastroenterol Hepatol.

[4] Ingle SB, Hinge Ingle CR (2013). Eosinophilic gastroenteritis: an unusual type of gastroenteritis. World J Gastroenterol.

[5] Zhang M, Li Y (2017). Eosinophilic gastroenteritis: a state-of-the-art review. J Gastroenterol Hepatol.

[6] Pinte L, Baicuş C (2019). Causes of eosinophilic ascites - a systematic review. Rom J Intern Med.

[7] Cello J (1979). Eosinophilic gastroenteritis: a complex disease entity. Am J Med.

[8] Klein N, Hargrove R, Sleisenger M, Jeffries G (1970). Eosinophilic gastroenteritis. Medicine (Baltimore).

[9] Rothenberg ME (2004). Eosinophilic gastrointestinal disorders (EGID). J Allergy Clin Immunol.

[10] Lucendo AJ (2010). Eosinophilic diseases of the gastrointestinal tract. Scand J Gastroenterol.

[11] Abou Rached A, El Hajj W (2016). Eosinophilic gastroenteritis: approach to diagnosis and management. World J Gastrointest Pharmacol Ther.

[12] Dellon ES (2012). Diagnosis and management of eosinophilic esophagitis. Clin Gastroenterol Hepatol.

[13] Nicoll R, Fortune J, MacDonald JC, Swann R (2023). Eosinophilic gastroenteritis: an unusual cause of vomiting and diarrhoea. BMJ Case Rep.

[14] Chen MJ, Chu CH, Lin SC, Shih SC, Wang TE (2003). Eosinophilic gastroenteritis: clinical experience with 15 patients. World J Gastroenterol.

[15] Mishra A, Hogan SP, Brandt EB, Rothenberg ME (2001). An etiological role for aeroallergens and eosinophils in experimental esophagitis. J Clin Invest.

[16] Ramma W, Ahmed A (2011). Is inflammation the cause of pre-eclampsia?. Biochem Soc Trans.

[17] Redman CW, Sargent IL (2005). Latest advances in understanding preeclampsia. Science.

[18] Ribeiro MI, Cardoso N, Pires S, Veloso T, Barata C (2018). Post-partum eosinophilic gastroenteritis: a case report. Gastroenterol Hepatol.

[19] Khalil H, Joseph M (2016). Eosinophilic ascites: a diagnostic challenge. BMJ Case Rep.

